# Distinct roles of nutritional and inflammatory signatures in predicting pathological response versus long-term survival in locally advanced gastric cancer treated with neoadjuvant immunotherapy

**DOI:** 10.3389/fonc.2026.1774606

**Published:** 2026-06-18

**Authors:** Ying Dong, Lei Qian, YiChong Wang, Chunzhi Yuan, Wenjing Cui, Yue Shi, Jun Zhu, Xiaochen Ding

**Affiliations:** 1 Department of Laboratory, The Affiliated Hospital of Northwest University, Xi’an No.3 Hospital, Xi’an, China; 2 Department of Experimental Surgery, Xijing Hospital, The First Affiliated Hospital of Fourth Military Medical University, Xi’an, China; 3 Department of General Surgery, The Southern Theater Air Force Hospital, Guangzhou, China; 4 Department of Pathology, Xijing Hospital, The First Affiliated Hospital of Fourth Military Medical University, Xi’an, China; 5 Department of Pathology, Xi’an Children’s Hospital, Xi’an, China

**Keywords:** creatinine-to-cystatin C ratio, gastric cancer, immunotherapy, neoadjuvant therapy, prognostic indicators, prognostic nutritional index, systemic immune-inflammation index

## Abstract

**Background:**

Neoadjuvant immunotherapy combined with chemotherapy has revolutionized the treatment of locally advanced gastric cancer (LAGC), yet optimizing patient selection remains challenging due to heterogeneous responses. This study aimed to evaluate the divergent predictive values of nutritional and inflammatory biomarkers—specifically the Prognostic Nutritional Index (PNI), Systemic Immune-inflammation Index (SII), and Creatinine to Cystatin C Ratio (CCR)—for short-term pathological response and long-term survival.

**Methods:**

A prospective cohort of 132 LAGC patients receiving neoadjuvant nivolumab plus chemotherapy followed by curative resection was analyzed. Logistic regression and a decision tree model were employed to identify predictors of pathological complete response (pCR). Furthermore, univariate and multivariate Cox proportional hazards regression analyses were performed to determine independent prognostic factors for overall survival (OS).

**Results:**

In this study, patients with pCR showed significantly better OS and disease-free survival (DFS) compared to patients with non-pCR (both P < 0.01). Additionally, patients in the pCR group exhibited a significant decrease in the SII levels, while PNI and CCR were significantly increased (all P < 0.05). Restricted Cubic Spline (RCS) analysis confirmed that these indices were significantly linearly correlated with survival risks (all P < 0.05), and Kaplan-Meier curves revealed that patients with low SII, high PNI and CCR levels had longer OS and DFS (all P < 0.0001). Based on decision tree analysis, a prediction model was constructed by combining PNI, SII, CCR and CA199, which significantly enhanced the predictive ability of pCR (Area Under the Curve, AUC = 0.917). Finally, the nomogram models for OS and DFS also demonstrated good calibration and discrimination.

**Conclusions:**

Nutritional and inflammatory status (SII, PNI, CCR) and CA199 serve as a sensitive marker for immediate pathological response, SII, PNI and CA125 are more indicative of long-term prognosis. Integrating these distinct biomarkers into phase-specific predictive models facilitates precise risk stratification and personalized management for patients undergoing neoadjuvant immunotherapy.

## Introduction

Gastric cancer (GC) represents a major global health burden, accounting for a substantial proportion of cancer-associated deaths due to its persistently high incidence and mortality (1).In the management of locally advanced gastric cancer (LAGC), neoadjuvant chemotherapy (NAC) is widely adopted as a standard approach with the objectives of reducing tumor stage, increasing the likelihood of R0 resection, and ultimately improving patient survival (2, 3). In recent years, the combination of immune checkpoint inhibitors (ICIs) with traditional NAC has been confirmed in multiple studies to significantly increase the rate of pathological complete response (pCR) and better survival benefits for patients (4, 5). pCR has become a key alternative endpoint for evaluating the efficacy of NAC combined with immunotherapy.

Nevertheless, therapeutic outcomes show considerable interpatient variability (6), making reliable biomarkers crucial for personalized therapy to maximize benefit and minimize toxicity (7). Current research primarily focuses on tumor-specific biomarkers, such as PD-L1 expression, microsatellite instability (MSI) status, and tumor mutational burden (TMB) (8–10). Although these biomarkers hold certain predictive value, their application is still limited, often requiring invasive tumor tissue sampling.

The nutritional and inflammatory status of cancer patients plays a key role in tumorigenesis, tumor progression, and therapeutic responsiveness (11). Accumulating evidence indicates that malnutrition and inflammation synergistically generate an immunosuppressive microenvironment that promotes tumor proliferation, angiogenesis, and metastatic dissemination while simultaneously weakening anti-tumor immune responses (12, 13). The Prognostic Nutritional Index (PNI) integrates serum albumin levels and peripheral blood lymphocyte count, reflecting the patient’s nutritional status and immune adaptability (14). The Systemic Immune-inflammation Index (SII) combines neutrophil, platelet, and lymphocyte counts to dynamically characterize the systemic inflammatory state and immune balance (15). The Creatinine-to-Cystatin C Ratio (CCR) assesses the body’s muscle reserves and inflammation levels through the ratio of creatinine to cystatin C, and has recently been linked to survival and treatment response in cancer patients (16). PNI, SII, and CCR integrate parameters from peripheral blood lymphocytes, neutrophils, platelets, and albumin, enabling a comprehensive quantification of the patient’s nutritional status and systemic inflammation levels (17–19).

These biomarkers have been validated as strong prognostic predictors in various solid tumors. However, the predictive value of these easily accessible, low-cost biomarkers has yet to be systematically explored in GC undergoing NAC combined with immunotherapy.

Therefore, this study aims to comprehensively evaluate the predictive efficacy of the PNI, SII, CCR, and other related indicators in this specific patient population. The goal is to develop an effective predictive model that provides new, easily implementable tools and theoretical support for clinical decision-making.

## Method

### Patients and follow-up

This analysis enrolled patients diagnosed with LAGC who were administered neoadjuvant Nivolumab in combination with chemotherapy at Xijing Hospital from February 2021 to December 2024, followed by curative-intent surgical resection. Inclusion criteria were: (1) histologically confirmed GC; (2) clinical staging of cT3-4a, Whether the lymph nodes are positive or not, and M0 (according to the AJCC 8th edition); (3) completion of at least two cycles of neoadjuvant immunotherapy combined with chemotherapy; (4) availability of complete baseline peripheral blood test data prior to neoadjuvant therapy. Exclusion criteria included: (1) distant metastasis (M1); (2) presence of other malignant tumors; (3) incomplete clinical data. Patients were followed postoperatively, with clinical assessments scheduled at 3-month intervals in the first year and every 6 months subsequently. This study was approved by the Ethics Committee of Xijing Hospital (approval number: KY20222082-C-1), and the requirement for informed consent from patients was waived.

All patients received neoadjuvant immunotherapy combined with chemotherapy consisting of nivolumab plus XELOX (capecitabine plus oxaliplatin) or SOX (S-1 plus oxaliplatin) regimen. Nivolumab was administered intravenously at a fixed dose of 360 mg every 3 weeks prior to surgery. The specific regimen selection was determined by the treating physician based on patient performance status, comorbidities, and institutional protocol. Patients underwent radiological assessment every 2 cycles to evaluate treatment response, and surgery was scheduled 4–6 weeks after completion of the last neoadjuvant cycle.

### Data collection and variable definition

Patient data and laboratory parameters, including age, sex, neutrophil count, lymphocyte count, and platelet count, were collected through the electronic medical record system. Pathological complete response (pCR) is defined as the absence of viable tumor cells in both the primary tumor and lymph node specimens after surgery (TRG grade 0). Best overall response (BOR) was assessed according to the RECIST 1.1 criteria, categorized into complete response (CR), partial response (PR), stable disease (SD), and progressive disease (PD) (20). Overall survival (OS) is defined as the time from the start of treatment to death from any cause. Disease-free survival (DFS) is the time from surgery to disease recurrence, metastasis, or death from any cause.

The median follow-up duration was 20 months (range: 18–22 months). Disease recurrence was assessed by contrast-enhanced computed tomography (CT) of the chest, abdomen, and pelvis at each scheduled follow-up visit. Magnetic resonance imaging (MRI) or positron emission tomography-computed tomography (PET-CT) was additionally performed when clinically indicated to confirm suspected recurrence or metastasis. Recurrence was defined as radiological evidence of local tumor recurrence, regional lymph node metastasis, or distant metastasis, and was confirmed by two independent radiologists.

### Statistical analysis

All statistical analyses were performed using R software (version 5.1.0) and the relevant packages (`survival`, `rms`, `timeROC`, `ggplot2`). Categorical variables were presented as frequencies (percentages), and group comparisons were conducted using the chi-square test or Fisher’s exact test. Continuous variables that followed a normal distribution were expressed as means ± standard deviation (SD) and compared using the t-test. If not normally distributed, data were presented as medians (interquartile range) and comparisons were performed using the Mann-Whitney U test or Kruskal-Wallis test. Kaplan-Meier survival curves were generated, and group comparisons were made using the Log-rank test. Cox proportional hazards regression models were used to calculate hazard ratios (HR) and their 95% confidence intervals (CI). Restricted cubic splines (RCS) were employed to fit Cox models and validate the linear relationship between continuous variables and survival outcomes. The Shapley Additive Explanations (SHAP) values were used to interpret the contribution of each feature to individual predictions in machine learning models, such as the XGBoost-based survival model, thereby enhancing the model’s interpretability. Calibration curves and concordance index (C-index) were used to assess the predictive accuracy and discriminatory ability of nomograms.

## Result

### Patient baseline characteristics

A total of 132 patients with locally advanced gastric cancer who received neoadjuvant immunotherapy combined with chemotherapy were included in this study. The baseline clinical and pathological characteristics of all patients are summarized in **Table 1**. The mean age of the overall cohort was 59.8 ± 11 years, with 71 male patients (53.8%) and 61 female patients (46.2%). Among the patients, 29 (22.0%) achieved pathological complete response (pCR), and 48 (36.4%) achieved major pathological response (MPR).

**Table 1:** Baseline information for all patients.

	[ALL]	non-pCR	pCR	P value
	*N = 132*	*N = 103*	*N = 29*	
Age	59.8 ± 11	60.0 ± 10.5	59.0 ± 11	0.671
Gender:				1.000
female	61 (46.2%)	48 (46.6%)	13 (44.8%)	
male	71 (53.8%)	55 (53.4%)	16 (55.2%)	
TRG:				<0.001
0	29 (22.0%)	0 (0.00%)	29 (100%)	
1	31 (23.5%)	31 (30.1%)	0 (0.00%)	
2	38 (28.8%)	38 (36.9%)	0 (0.00%)	
3	34 (25.8%)	34 (33.0%)	0 (0.00%)	
CPS:				<0.001
<1	74 (56.1%)	67 (65.0%)	7 (24.1%)	
≥1	58 (43.9%)	36 (35.0%)	22 (75.9%)	
MPR:				<0.001
0	84 (63.6%)	84 (81.6%)	0 (0.00%)	
1	48 (36.4%)	19 (18.4%)	29 (100%)	
BOR:				<0.001
CR	29 (22.0%)	0 (0.00%)	29 (100%)	
PR	37 (28.0%)	37 (35.9%)	0 (0.00%)	
SD	47 (35.6%)	47 (45.6%)	0 (0.00%)	
PD	19 (14.4%)	19 (18.4%)	0 (0.00%)	
ypTNM:				<0.001
Stage I	56 (42.4%)	32 (31.1%)	24 (82.8%)	
Stage II	44 (33.3%)	39 (37.9%)	5 (17.2%)	
Stage III	32 (24.2%)	32 (31.1%)	0 (0.00%)	
Perineural invasion:				1.000
0	112 (84.8%)	87 (84.5%)	25 (86.2%)	
1	20 (15.2%)	16 (15.5%)	4 (13.8%)	
Vascular invasion:				0.964
0	102 (77.3%)	79 (76.7%)	23 (79.3%)	
1	30 (22.7%)	24 (23.3%)	6 (20.7%)	
ORR:				<0.001
0	66 (50.0%)	66 (64.1%)	0 (0.00%)	
1	66 (50.0%)	37 (35.9%)	29 (100%)	
DCR:				0.013
0	19 (14.4%)	19 (18.4%)	0 (0.00%)	
1	113 (85.6%)	84 (81.6%)	29 (100%)	
Neoadjuvant chemotherapy:				0.853
FLOT	68 (51.5%)	54 (52.4%)	14 (48.3%)	
SOX	64 (48.5%)	49 (47.6%)	15 (51.7%)	
SII	1253 [674;1749]	1419 [884;1912]	537 [435;770]	<0.001
PNI	39.2 [34.7;45.8]	37.5 [33.4;42.3]	46.6 [42.9;48.6]	<0.001
AFP	3.03 [2.15;4.03]	2.91 [2.05;3.98]	3.36 [2.43;4.12]	0.183
CEA	3.84 [2.12;12.0]	7.40 [2.58;13.0]	2.12 [1.58;2.94]	<0.001
CA199	11.4 [7.13;31.5]	12.4 [11.96;30.7]	9.38 [7.71;12.3]	0.005
CA125	11.4 [7.56;15.7]	10.8 [7.58;15.3]	11.5 [7.59;16.1]	0.765
CCR	119 (33.9)	113 (31.9)	142 (30.6)	<0.001

TRG, Tumor Regression Grade; CR, Complete Response; PR, Partial Response; SD, Stable Disease; PD, Progressive Disease; CPS, Combined Positive Score; MPR, Major Pathological Response; BOR, Best Overall Response; ORR, Objective Response Rate; DCR, Disease Control Rate; SII, Systemic Immune-Inflammation Index; PNI, Prognostic Nutritional Index; NLR, Neutrophil-to-Lymphocyte Ratio; AFP, Alpha-Fetoprotein; CEA, Carcinoembryonic Antigen; CA199, Carbohydrate Antigen 19-9; CA125, Cancer Antigen 125; CCR, creatinine to cystatin C ratio.

### Analysis of factors related to complete pathological remission

To explore the differences between patients who achieved pCR and non-pCR, this study compared the baseline characteristics of the pCR group (n=29) and the non-pCR group (n=103, **Table 1**). The analysis revealed no significant differences between the two groups in terms of age, gender, AFP, CA125, neural invasion, vascular invasion and distribution of neoadjuvant chemotherapy regimens (P > 0.05). However, several indicators closely associated with inflammation and nutritional status showed significant differences between the two groups. Patients who achieved pCR had lower baseline Carcinoembryonic Antigen (CEA), SII, and CA199 levels. while nutritional indicators (PNI and CCR) were significantly higher (P < 0.001).

### Survival analysis

**Figure 1:**
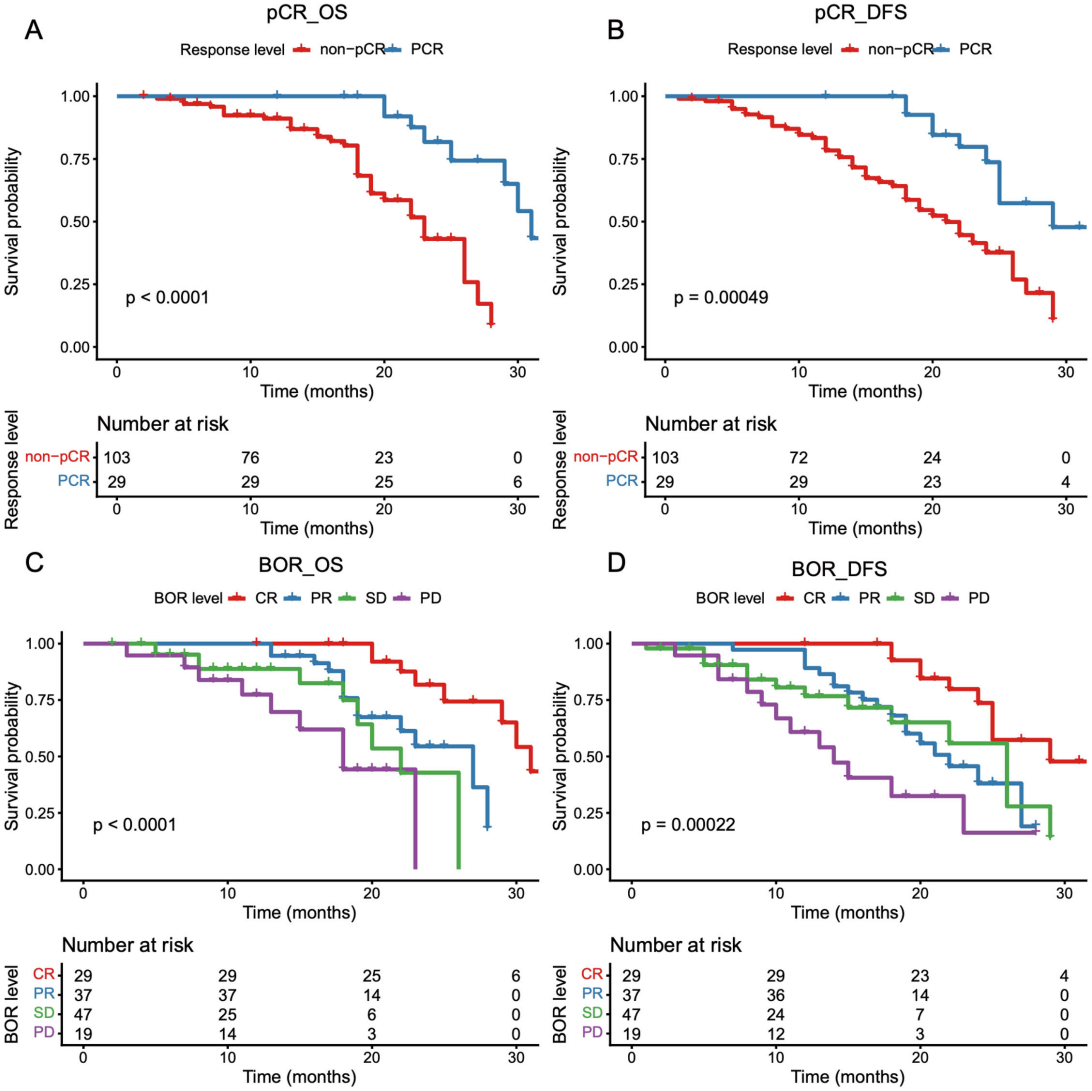
Kaplan-Meier survival curves of OS and DFS. **(A)**, OS based on pCR status; **(B)**, DFS based on pCR status; **(C)**, OS based on BOR; **(D)**, DFS based on BOR. OS, Overall Survival; DFS, Disease-Free Survival; BOR, Best Overall Response; pCR, Pathological Complete Response.

Subsequently, this study employed Kaplan-Meier (KM) survival analysis to explore the correlation between pCR and survival outcomes. Survival analyses revealed markedly superior OS and DFS in patients attaining pCR relative to non-pCR (Log-rank P < 0.0001; **Figures 1A, B**). (Log-rank P < 0.0001, **Figures 1A, B**). Similarly, based on BOR grouping, patients with CR had the best prognosis, followed by those with PR and SD, while patients with PD had the worst prognosis. The differences in OS and DFS between the groups were statistically significant (all Log-rank P < 0.0001, **Figures 1C, D**).

### Association between inflammatory/nutritional markers and treatment prognosis

**Figure 2:**
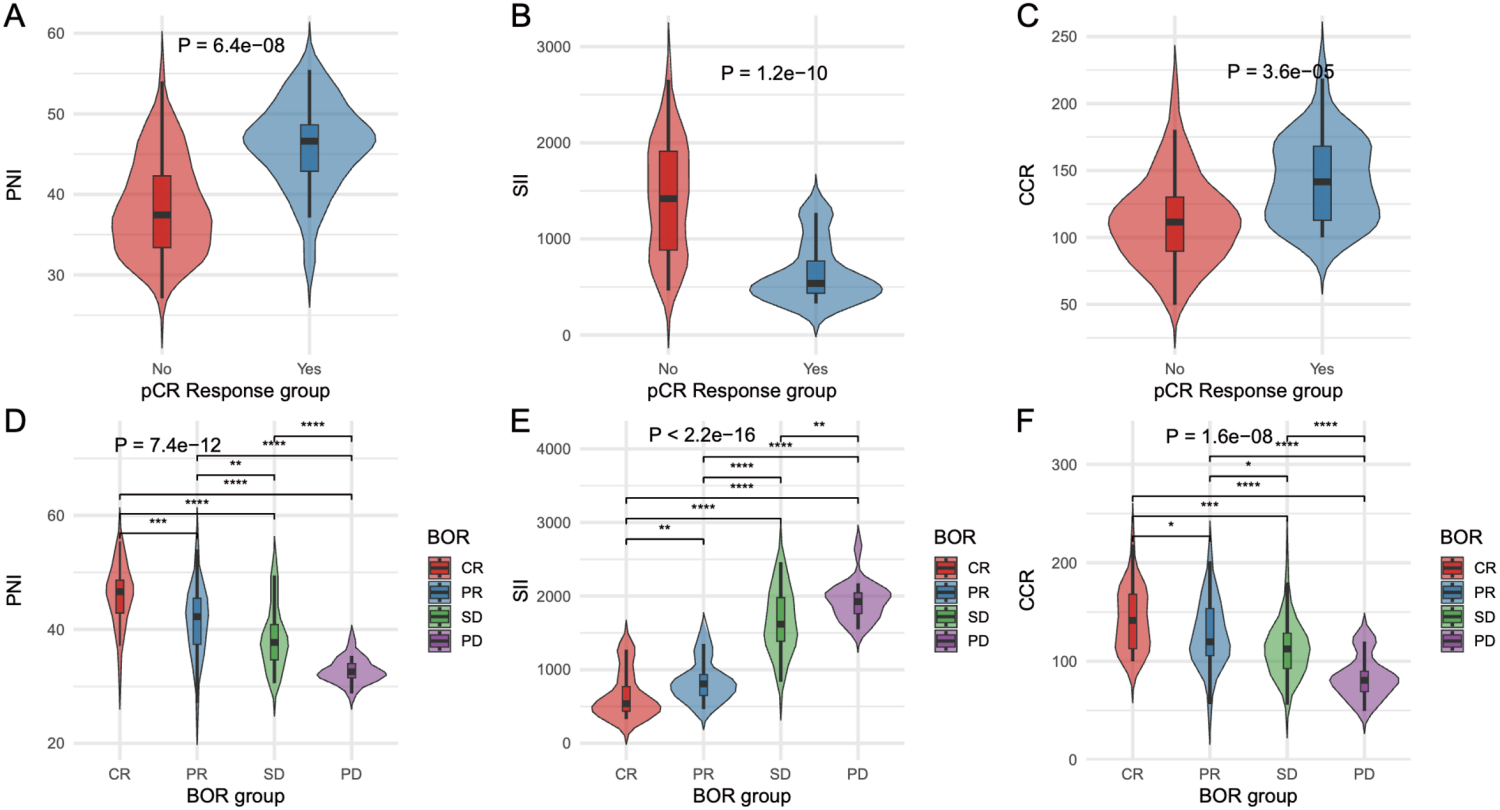
Comparison of baseline biomarkers (PNI, SII, CCR) across response groups. **(A)**, Comparison of PNI between pCR and non-pCR groups; **(B)**, Comparison of SII between pCR and non-pCR groups; **(C)**, Comparison of CCR between pCR and non-pCR groups; **(D)**, Comparison of PNI according to BOR (CR, PR, SD, PD); **(E)**, SII distribution across BOR groups; **(F)**, CCR distribution across BOR groups. SII, Systemic Immune-Inflammation Index; PNI, Prognostic Nutritional Index; CCR, creatinine to cystatin C ratio; pCR, Pathological Complete Response; BOR, Best Overall Response; CR, Complete Response; PR, Partial Response; SD, Stable Disease; PD, Progressive Disease. Values represent statistical significance: *P < 0.05, **P < 0.01, *P < 0.001, **P < 0.0001.

Additionally, this study explored the relationship between pretreatment SII, PNI, and CCR and the efficacy of neoadjuvant immunotherapy combined with chemotherapy. Compared with patients without pCR, those with pCR had lower SII levels, but higher PNI and CCR levels (**Figures 2A–C**; all P < 0.05). Similarly, in the BOR assessment, patients with better responses (CR/PR) showed lower SII levels, but higher PNI and CCR levels than those with worse response (SD/PD) (**Figures 2D–F**; all P < 0.05).

**Figure 3:**
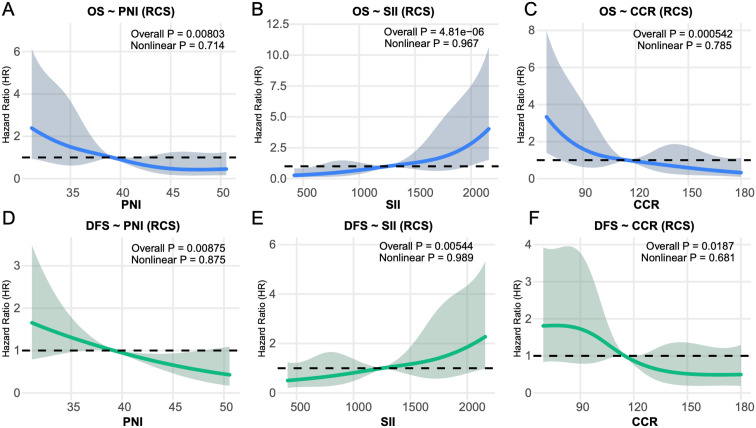
Restricted cubic spline (RCS) analysis of survival risk. **(A)**, Association between PNI and OS; **(B)**, Association between SII and OS; **(C)**, Association between CCR and OS; **(D)**. Association between PNI and DFS; **(E)**. Association between SII and DFS; **(F)**. Association between CCR and DFS. SII, Systemic Immune-Inflammation Index; PNI, Prognostic Nutritional Index; CCR, creatinine to cystatin C ratio; pCR, Pathological Complete Response; OS, overall survival; DFS, Disease-Free Survival.

Furthermore, to further validate the association between these continuous variables and survival risk, we performed RCS analysis. The results indicated that PNI, SII, and CCR were significantly associated with both OS and DFS (all P < 0.05). RCS analysis showed a linear relationship between PNI, SII, and CCR levels and the probability of pCR (P for nonlinearity > 0.05), supporting their use as continuous variables in prognostic models (**Figure 3**).

Univariate and multivariate logistic regression analyses were performed to identify predictors of pCR (**Table 2**). In univariate analysis, lower SII (OR 0.99, 95% CI 0.99–1.00, P<0.001), higher PNI (OR 1.25, 95% CI 1.15–1.39, P<0.001), higher CCR (OR 1.03, 95% CI 1.01–1.04, P<0.001), lower CEA levels (OR 0.98, 95% CI 0.97–0.99, P = 0.004), lower CA199 levels (OR 0.97, 95% CI 0.95–0.99, P = 0.009), and stage II disease (OR 0.17, 95% CI 0.05–0.47, P = 0.001) were significantly associated with pCR. Multivariate analysis revealed that lower SII (OR 0.99, 95% CI 0.99–1.00, P = 0.032), higher PNI (OR 1.15, 95% CI 1.02–1.33, P = 0.028), higher CCR (OR 1.02, 95% CI 1.01–1.03, P = 0.017), and stage II disease (OR 0.14, 95% CI 0.03–0.56, P = 0.009) remained independent predictors of pCR.

**Table 2:** Univariate and multivariate logistic regression analysis of predictors of pathological complete response.

Characteristic	Univariate analysis			Multivariate analysis		
	OR	95% CI	P value	OR	95% CI	P value
SII	0.99	0.99, 1.00	**<0.001**	0.99	0.99, 1.00	**0.032**
PNI	1.25	1.15, 1.39	**<0.001**	1.15	1.02, 1.33	**0.028**
CCR	1.03	1.01, 1.04	**<0.001**	1.02	1.01, 1.03	**0.017**
CEA	0.98	0.97, 0.99	**0.004**	0.77	0.50, 0.99	0.123
CA199	0.97	0.95, 0.99	**0.009**	0.98	0.96, 0.99	**0.030**
CA125	1.00	0.97, 1.03	0.829			
ypTNM						
Stage I	—	—	ref	—	—	ref
Stage II	0.17	0.05, 0.47	**0.001**	0.14	0.03, 0.56	**0.009**
Stage III	0.00	0.00, NA	0.987			
gender						
female	—	—	ref			
male	1.07	0.47, 2.49	0.866			
Neoadjuvant chemotherapy						
FLOT	—	—	ref			
SOX	1.18	0.52, 2.72	0.693			

CI, Confidence Interval; OR, Odds Ratio; SII, Systemic Immune-Inflammation Index; PNI, Prognostic Nutritional Index; CCR, creatinine to cystatin C ratio; CA199, Carbohydrate Antigen 19-9; CA125, Cancer Antigen 125.

Bold font indicates P < 0.05.

**Figure 4:**
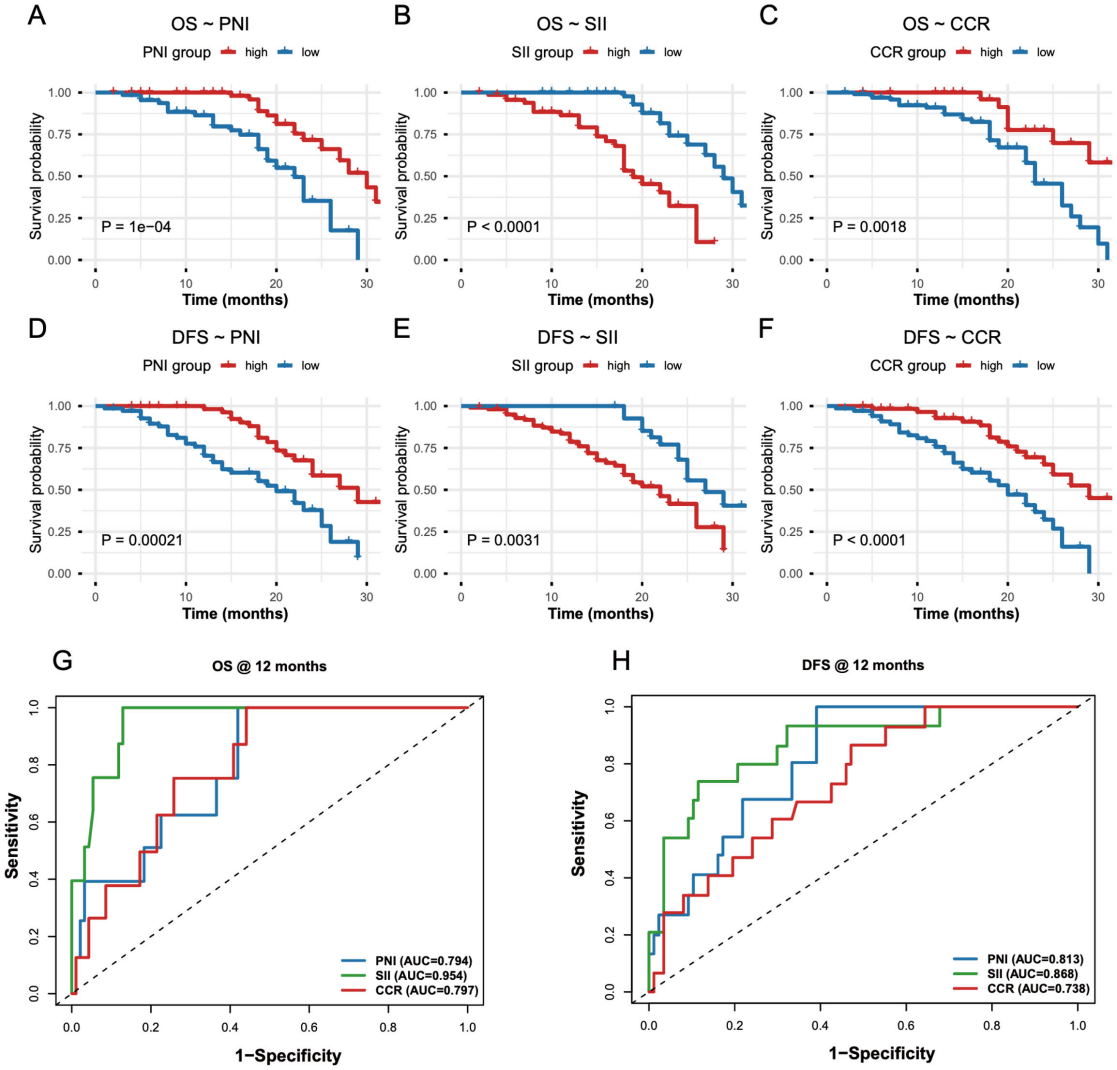
Kaplan-Meier survival curves and ROC analysis for prognostic biomarkers. **(A)**,: OS stratified by PNI (high vs. low); **(B)**, OS stratified by SII (high vs. low); **(C)**, OS stratified by CCR (high vs. low); **(D)**, DFS stratified by PNI (high vs. low); **(E)**, DFS stratified by SII (high vs. low); **(F)**, DFS stratified by CCR (high vs. low); **(G–L)**, Time-dependent ROC curves for OS and DFS prediction at 12-month after surgery. SII, Systemic Immune-Inflammation Index; PNI, Prognostic Nutritional Index; CCR, creatinine to cystatin C ratio; OS, Overall Survival; DFS, disease-free survival; ROC, Receiver Operating Characteristic.

Subsequently, based on the optimal cutoff values determined through RCS curves, patients were divided into high or low PNI groups, high or low SII groups, and high or low CCR groups. Regarding overall survival (OS), patients with high PNI exhibited better survival outcomes than those with low PNI (P < 0.0001 for both, **Figure 4A**). In contrast, patients with low SII had superior OS compared to those with high SII (P < 0.0001 for both, **Figure 4B**). Patients in the low CCR group had significantly poorer OS, which was substantially lower than that of the high CCR group (P < 0.0001 for both, **Figure 4C**). A similar trend was observed in disease-free survival (DFS), where high PNI, low SII, and high CCR were all significantly associated with prolonged DFS (P < 0.0001 for all, **Figures 4D–F**).

Based on ROC curve analysis, we further evaluated the prognostic performance of PNI, SII, and CCR at different time points for OS. As shown in **Figure 4G**, for 12-month OS prediction, SII demonstrated the highest discriminatory ability with an AUC of 0.954, significantly outperforming PNI (AUC = 0.794) and CCR (AUC = 0.797). At 18 months, the predictive power of SII exhibited the optimal discriminatory ability (AUC = 0.899), while the AUC for PNI increased to 0.781 and for CCR to 0.765. In the 24-month OS prediction, SII continued to show the best performance (AUC = 0.825), with PNI and CCR having AUC values of 0.751 and 0.751, respectively (**Supplementary Figures 1A, B**). In DFS prediction, the performance of each indicator showed a time-dependent change: at 12 months, SII remained superior (AUC = 0.868) than PNI (AUC = 0.813) and CCR (AUC = 0.738; **Figure 4H**). At 18 months, the AUC values for SII, PNI, and CCR were 0.834, 0.734, and 0.727, respectively (**Supplementary Figure 1C**). At 24 months, the corresponding AUC values were 0.717, 0.629, and 0.691 (**Supplementary Figure 1D**).

### Construction and evaluation the prediction model

**Figure 5:**
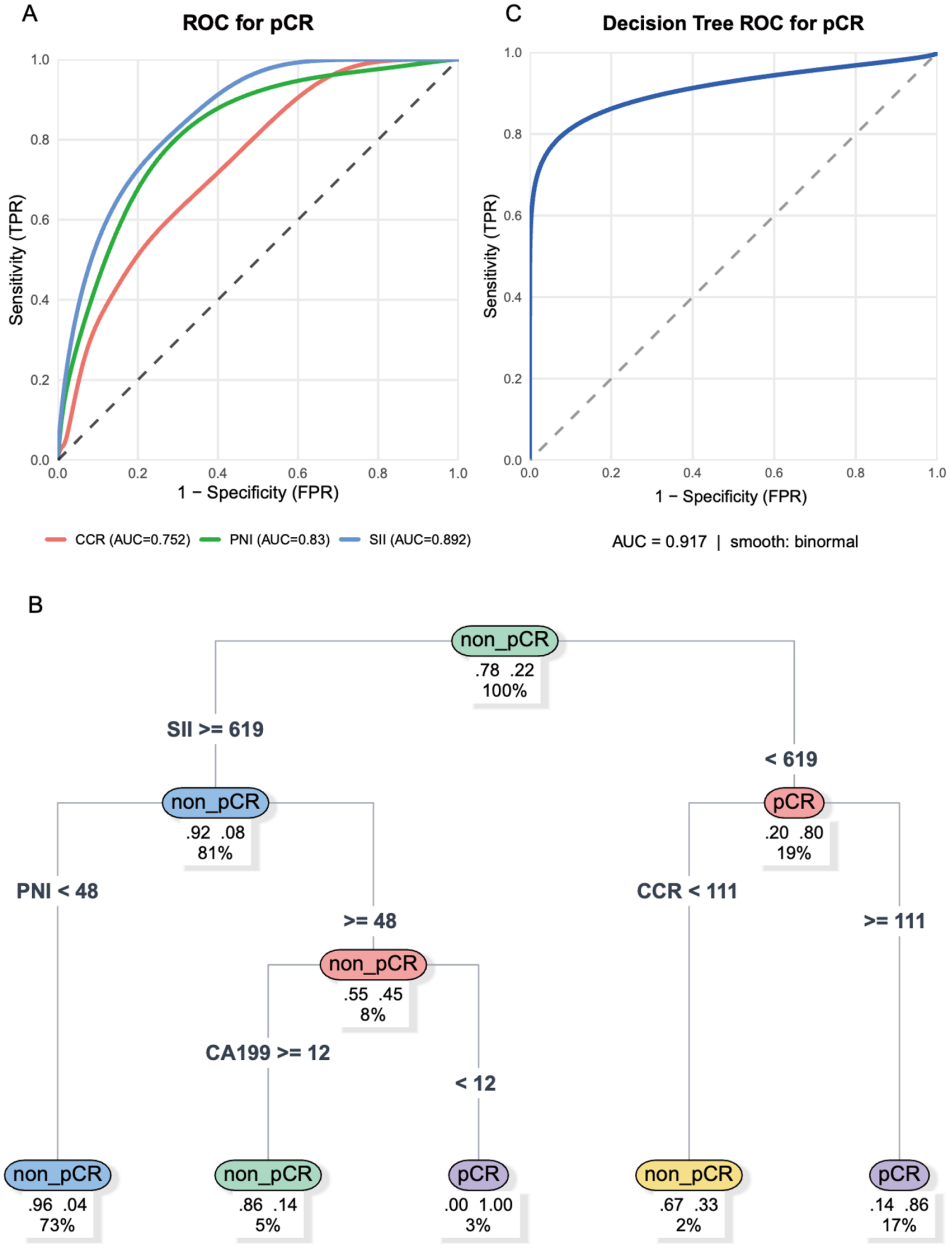
Predictive accuracy of biomarkers and decision tree model. **(A)**, ROC analysis of single biomarkers for predicting pCR; **(B)**, ROC curve of decision tree model integrating SII, PNI, and CCR; **(C)**, Visualization of the decision tree classification. SII, Systemic Immune-Inflammation Index; PNI, Prognostic Nutritional Index; CCR, creatinine to cystatin C ratio; ROC, Receiver Operating Characteristic.

Subsequently, to predict the efficacy of neoadjuvant immunotherapy combined with chemotherapy in patients, we plotted ROC curves. As shown in **Figure 5**, SII demonstrated excellent predictive performance, with an AUC of 0.892, significantly outperforming PNI (AUC = 0.83) and CCR (AUC = 0.752). We then constructed a decision tree model using the differential characteristics between the pCR and non-pCR groups (CEA, PNI, SII, and CCR). To facilitate clinical decision-making, the decision tree model was visualized (**Figure 5B**). The AUC the decision tree model was 0.984, indicating good predictive ability (**Figure 5C**).

**Figure 6:**
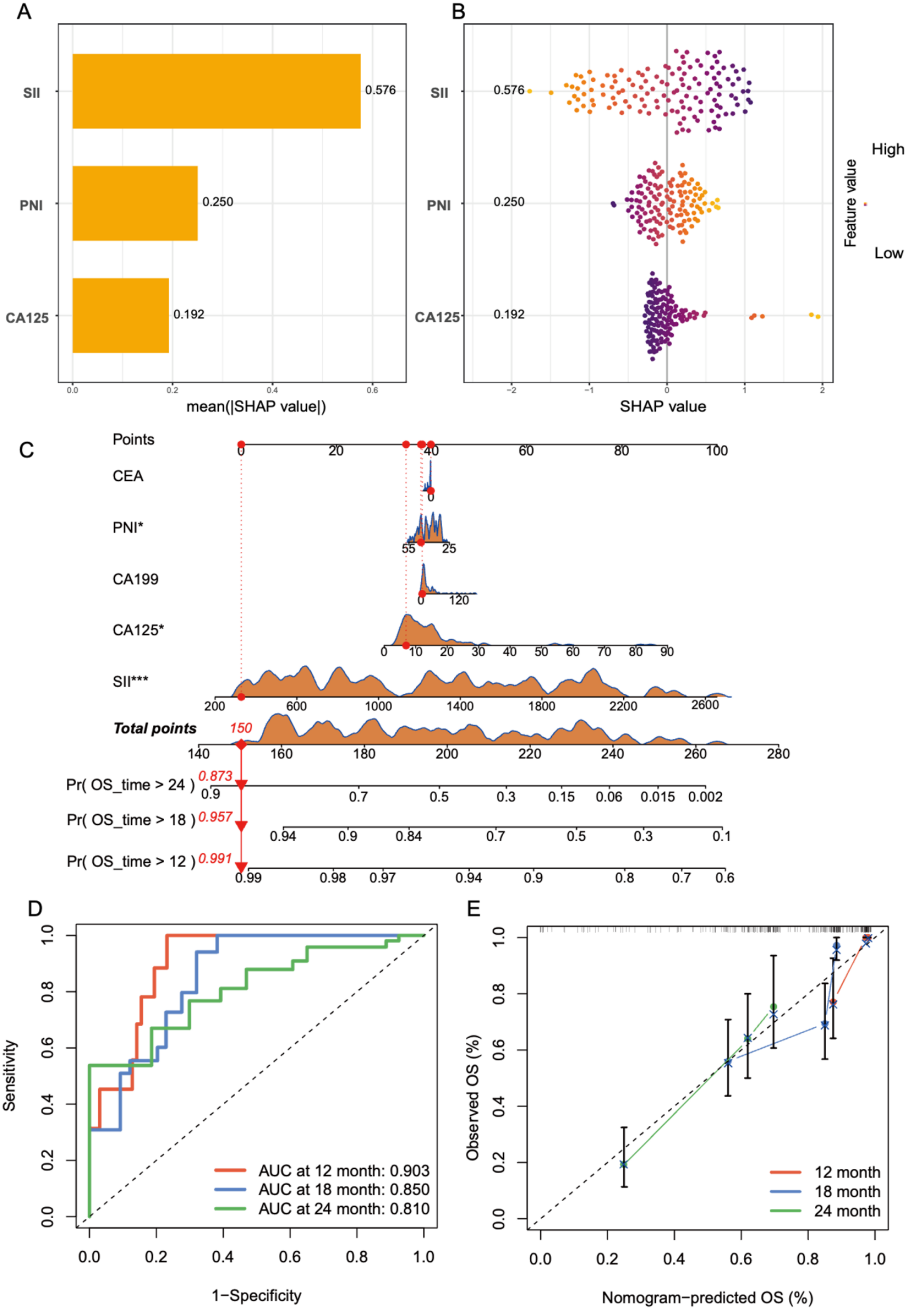
Construction of prognostic nomogram and model evaluation. **(A)**, SHAP summary plot ranking feature importance for survival prediction. **(B)**, SHAP dependence plot showing feature effect on OS; **(C)**, Nomogram integrating SII, PNI, CEA, and CA125 for predicting 12-, 18-, and 24-month OS probabilities; **(D)**, ROC curves demonstrating good discrimination of the nomogram; **(E)**, Calibration plots showing good agreement between predicted and observed OS at different time points. SHAP, Shapley Additive Explanations; SII, Systemic Immune-Inflammation Index; PNI: Prognostic Nutritional Index; CCR, creatinine to cystatin C ratio; ROC, Receiver Operating Characteristic; OS, Overall Survival.

Finally, this study developed a prognostic model based on patient baseline indicators. To enhance the interpretability of the model, SHAP values were applied to analyze the contribution of each indicator to the patient’s prognosis. The results showed that SII was the most important predictive feature, followed by PNI and CA125 (**Figures 6A, B**). Then, we identified the prognostic factors by multivariate COX analysis (**Table 3**), and integrated the independent prognostic factors (CEA, PNI, SII, CA199 and CA125) to construct a nomogram for individualized prediction of OS probabilities at 12, 18, and 24 months (**Figure 6C**). This model demonstrated satisfactory discriminatory ability, with AUCs of 0.903, 0.850, and 0.810 at 12, 18, and 24 months, respectively (**Figure 6D**). The calibration curve showed good consistency between the predicted probabilities and actual observed outcomes (**Figure 6E**).

**Table 3:** Multivariate COX regression analysis of predictors of overall survival.

Characteristic	HR	95% CI	P value
SII	1.02	1.01, 1.03	**<0.001**
PNI	1.01	1.00, 1.02	**0.014**
CCR	0.99	0.98, 1.00	0.124
CEA	0.99	0.93, 1.05	0.053
CA199	1.00	0.99, 1.01	0.433
CA125	1.02	1.00, 1.04	**0.036**
ypTNM			
Stage I	—	—	
Stage II	2.66	1.10, 6.46	**0.030**
Stage III	1.29	0.45, 3.67	0.633
gender			
female	—	—	
male	1.22	0.59, 2.53	0.588
Neoadjuvant chemotherapy			
FLOT	—	—	
SOX	1.21	0.62, 2.37	0.575

CI, Confidence Interval; OR, Odds Ratio; SII, Systemic Immune-Inflammation Index; PNI, Prognostic Nutritional Index; CCR, creatinine to cystatin C ratio; CA199, Carbohydrate Antigen 19-9; CA125, Cancer Antigen 125.

Bold font indicates P < 0.05.

## Discussion

Through a prospective cohort analysis of LAGC patients receiving neoadjuvant immunotherapy, this investigation corroborates that the achievement of pCR is strongly associated with enhanced survival, which is highly consistent with the results from traditional chemotherapy and recent immunotherapy studies. This further validates pCR as a reliable surrogate endpoint for evaluating the efficacy of neoadjuvant treatments and predicting long-term survival. More importantly, even in patients who did not achieve pCR, the BOR showed a clear gradient relationship with survival outcomes (CR > PR > SD > PD). This indicates that, for patients who do not achieve pCR, the degree of tumor regression remains a critical indicator for evaluating treatment benefit and prognosis. Furthermore, this study highlights the significant prognostic value of systemic inflammatory and nutritional biomarkers, including the SII, PNI, and CCR. These indicators reflect the underlying host immune-nutritional status and systemic inflammation, which may modulate responses to immunotherapy and influence long-term survival outcomes.

The introduction of ICIs into the neoadjuvant treatment regimen for LAGC addresses key limitations of traditional chemotherapy, particularly in modulating the immunosuppressive tumor microenvironment (TME) (21, 22). Unlike cytotoxic drugs that primarily target rapidly dividing cells, ICIs reverse T-cell exhaustion and promote antigen-specific anti-tumor immunity (23). This immune remodeling is reflected in the increased pCR rates observed in recent trials, which are not only associated with tumor shrinkage but also with fundamental changes in the TME, such as increased CD8+ T-cell infiltration and reduced prevalence of regulatory T-cells (24). Furthermore, even in non-pCR patients, the gradient relationship between the BOR and survival suggests that the immune activation profile induced by ICIs extends beyond the binary “all-or-nothing” response. This underscores the potential of immunotherapy to transform “cold” tumors into “hot” tumors, thereby achieving long-term disease control (25).

Furthermore, this study reveals the critical impact of systemic inflammation and nutritional status on treatment efficacy before treatment. SII and CCR are reliable indicators reflecting systemic inflammation, while PNI simultaneously reflects both immune and nutritional status. These results suggest that patients with low inflammation and high immune-nutritional status pretreatment may experience better therapeutic outcomes. This is consistent with findings in various solid tumors, including liver cancer and colorectal cancer (26–28). On one hand, a high inflammatory state typically indicates an immunosuppressive microenvironment (such as one rich in myeloid-derived suppressor cells and M2 macrophages), which may diminish the activation effects of immune checkpoint inhibitors and promote tumor proliferation and metastasis (29, 30). On the other hand, a good nutritional and immune status is fundamental for maintaining effector T cell function and tissue repair capacity, which are critical for generating an effective anti-tumor immune response (31).

Through RCS analysis, we demonstrated a significant linear relationship between SII, PNI, and CCR and survival risk, rather than a simple binary relationship. This suggests that the clinical value of these indicators may extend beyond the application of a single cutoff value. This finding implies that, in clinical practice, evaluating these indicators as continuous variables could offer more refined risk stratification and provide a theoretical basis for future studies exploring the modulation of host inflammatory status to enhance the efficacy of immunotherapy (e.g., using anti-inflammatory drugs, nutritional support, etc.) (32, 33).

When exploring survival outcomes at different time points, various indicators exhibited differentiated value. SII showed the best predictive performance for survival, while PNI demonstrated greater stability in predicting medium to long term survival. This time dependent difference in predictive efficacy suggests that inflammation status may primarily affect short term treatment response and survival, while nutritional and immune status have a more lasting impact on long-term prognosis (34, 35).

Based on these findings, we successfully developed two tools with high clinical application potential. The decision tree model, utilizing SII, PNI, CA199 and CCR, enables high precision prediction of pCR through a clear logical process. This model assists in identifying the patient population most likely to benefit from intensive neoadjuvant immunotherapy prior to treatment, providing intuitive decision support for personalized treatment strategies.

Furthermore, the nomogram, which integrates multiple indicators, extends the prediction to long-term survival probabilities. Its excellent predictive performance makes it a promising practical tool for individualized prognostic assessment, aiding clinicians in facilitating more in-depth discussions with patients regarding adjuvant treatment intensity and follow-up plans.

An interesting finding in our study is the differential predictive value of biomarkers for short-term response (pCR) versus long-term survival (OS). The decision tree highlighted CCR as a key determinant for pCR, likely reflecting the impact of muscle mass and metabolic reserve on chemotherapy tolerance and immediate tumor killing. In contrast, the OS nomogram incorporated CA125, suggesting that tumor burden and specific biological behaviors might play a more dominant role in long-term disease progression and recurrence. This distinction underscores the importance of selecting outcome-specific biomarkers in clinical practice.

This study has several limitations. Firstly, it is a single-center study, which inevitably introduces selection bias. Secondly, the sample size is relatively small, particularly with a limited number of patients in the pCR subgroup, which may affect the statistical power of some analyses. However, despite these limitations, the data in this study are relatively robust for gastric cancer patients receiving neoadjuvant immunotherapy. Further validation is required through prospective cohort studies and basic experimental research, such as by analyzing the dynamic changes in the tumor immune microenvironment before and after treatment to confirm these findings.

## Conclusion

This study confirms that baseline nutritional and inflammatory status are key determinants of treatment response and long-term outcomes in gastric cancer patients undergoing neoadjuvant immunotherapy. The ideal baseline status for short-term efficacy may differ from that required for long-term survival, which deepens our understanding of the complexities of immunotherapy. Incorporating easily accessible indicators such as PNI and SII into clinical decision-making processes can help more accurately identify favorable populations, alert high-risk patients, and provide important theoretical support for future exploration of combined anti-inflammatory or nutritional intervention strategies to improve long-term survival outcomes for patients.

## Data Availability

The raw data supporting the conclusions of this article will be made available by the authors, without undue reservation.
