# Supplementary material for: Distinct roles of nutritional and inflammatory signatures in predicting pathological response versus long-term survival in locally advanced gastric cancer treated with neoadjuvant immunotherapy
Source: Front Oncol. 2026 Jun 18;16:1774606. doi: 10.3389/fonc.2026.1774606 (PMC13322926; doi:10.3389/fonc.2026.1774606)
Supplement: Supplementary file 1 [file DataSheet1.docx]

**Title: Distinct Roles of Nutritional and Inflammatory Signatures in Predicting Pathological Response versus Long-Term Survival in Locally Advanced Gastric Cancer Treated with Neoadjuvant Immunotherapy**


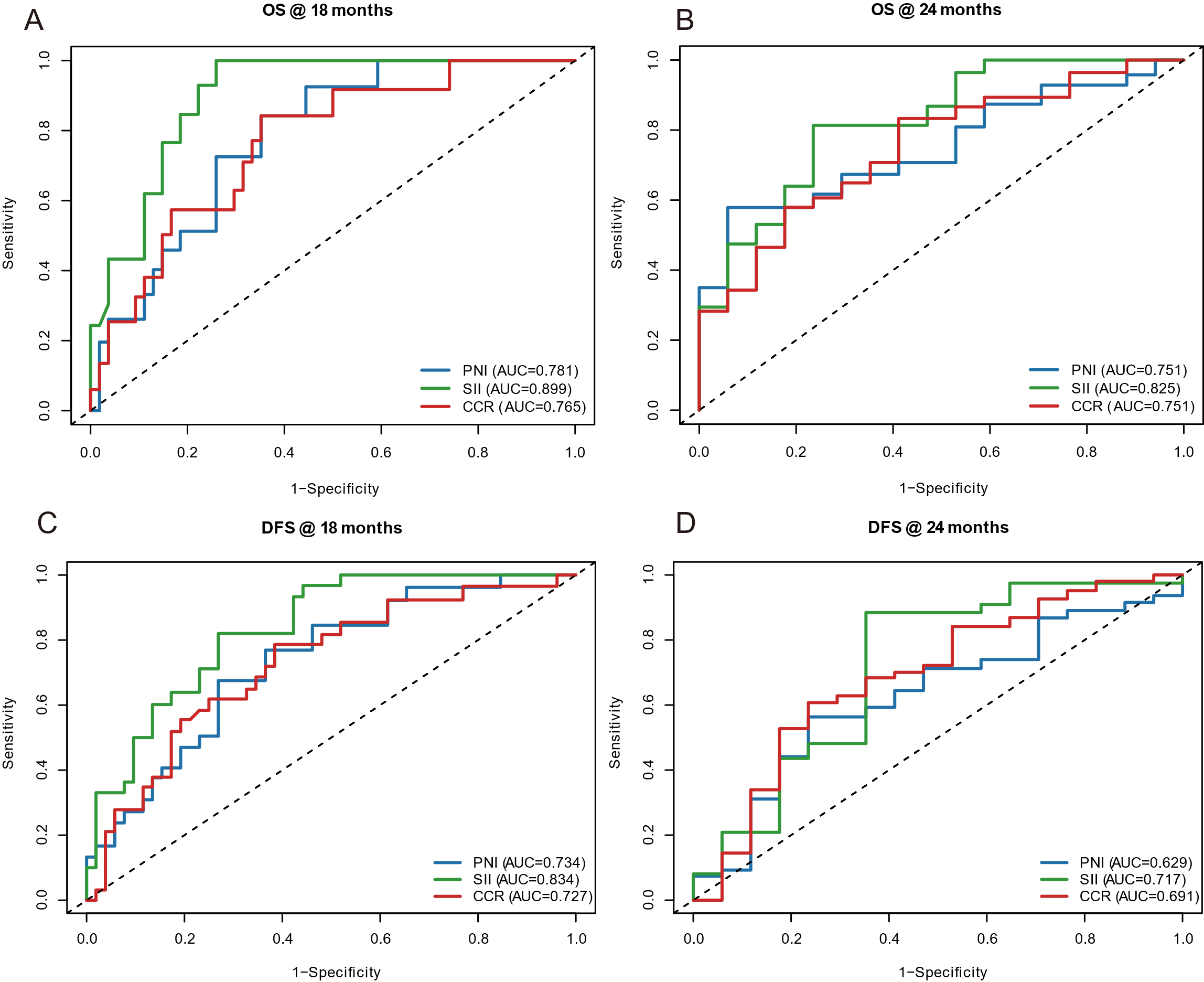


**Figure S1 The predictive value of PNI, SII and CCR for OS at 18- and 24- months.**

The ROC curves of PNI, SII and CCR for OS at 18- month OS (A), 24- months OS(B), 18- month DFS (C) and 24- month DFS (D) for locally advanced gastric cancer patients treated with neoadjuvant immunotherapy. SII: Systemic Immune-Inflammation Index; PNI: Prognostic Nutritional Index; CCR: creatinine to cystatin C ratio; OS, Overall Survival; DFS: disease-free survival; ROC: Receiver Operating Characteristic.
